# *Helicobacter pylori* VacA, acting through receptor protein tyrosine phosphatase α, is crucial for CagA phosphorylation in human duodenum carcinoma cell line AZ-521

**DOI:** 10.1242/dmm.025361

**Published:** 2016-12-01

**Authors:** Masayuki Nakano, Kinnosuke Yahiro, Eiki Yamasaki, Hisao Kurazono, Junko Akada, Yoshio Yamaoka, Takuro Niidome, Masanori Hatakeyama, Hidekazu Suzuki, Taro Yamamoto, Joel Moss, Hajime Isomoto, Toshiya Hirayama

**Affiliations:** 1 Department of Bacteriology, Institute of Tropical Medicine, Nagasaki University, 1-12-4, Sakamoto, Nagasaki 852-8523, Japan; 2 Department of International Health, Institute of Tropical Medicine, Nagasaki University, 1-12-4, Sakamoto, Nagasaki 852-8523, Japan; 3 Department of Molecular Infectiology, Graduate School of Medicine, Chiba University, 1-8-1, Inohana, Chuo-ku, Chiba 260-8670, Japan; 4 Division of Food Hygiene, Department of Animal and Food Hygiene, Obihiro University of Agriculture and Veterinary Medicine, Nishi 2-11, Inada-cho, Obihiro, Hokkaido 080-8555, Japan; 5 Department of Environmental and Preventive Medicine, Oita University Faculty of Medicine, Idaigaoka 1-1, Yufu, Oita 879-5593, Japan; 6 Department of Medicine, Gastroenterology and Hepatology Section, Baylor College of Medicine, Houston, TX 77030, USA; 7 Department of Applied Chemistry and Biochemistry, Graduate School of Science and Technology, Kumamoto University, 2-39-1 Kurokami, Chuo-ku, Kumamoto 860-8555, Japan; 8 Division of Microbiology, Graduate School of Medicine, The University of Tokyo, 7-3-1 Hongo, Bunkyo-Ku, Tokyo 113-0033, Japan; 9 Medical Education Center, Keio University School of Medicine, 35 Shinanomachi, Shinjuku-ku, Tokyo 160-8582, Japan; 10 Cardiovascular and Pulmonary Branch, NHLBI, National Institutes of Health, Bethesda, MD 20892-1590, USA; 11 Division of Medicine and Clinical Science, Tottori University Faculty of Medicine, 86 Nishi-cho, Yonago, Tottori 683-8503, Japan

**Keywords:** *Helicobacter pylori*, VacA, CagA

## Abstract

*Helicobacter pylori*, a major cause of gastroduodenal diseases, produces vacuolating cytotoxin (VacA) and cytotoxin-associated gene A (CagA), which seem to be involved in virulence. VacA exhibits pleiotropic actions in gastroduodenal disorders via its specific receptors. Recently, we found that VacA induced the phosphorylation of cellular Src kinase (Src) at Tyr418 in AZ-521 cells. Silencing of receptor protein tyrosine phosphatase (RPTP)α, a VacA receptor, reduced VacA-induced Src phosphorylation. Src is responsible for tyrosine phosphorylation of CagA at its Glu-Pro-Ile-Tyr-Ala (EPIYA) variant C (EPIYA-C) motif in *Helicobacter* *pylori*-infected gastric epithelial cells, resulting in binding of CagA to SHP-2 phosphatase. Challenging AZ-521 cells with wild-type *H. pylori* induced phosphorylation of CagA, but this did not occur when challenged with a *vacA* gene-disrupted mutant strain. CagA phosphorylation was observed in cells infected with a *vacA* gene-disrupted mutant strain after addition of purified VacA, suggesting that VacA is required for *H. pylori*-induced CagA phosphorylation. Following siRNA-mediated *RPTPα* knockdown in AZ-521 cells, infection with wild-type *H. pylori* and treatment with VacA did not induce CagA phosphorylation. Taken together, these results support our conclusion that VacA mediates CagA phosphorylation through RPTPα in AZ-521 cells. These data indicate the possibility that Src phosphorylation induced by VacA is mediated through RPTPα, resulting in activation of Src, leading to CagA phosphorylation at Tyr972 in AZ-521 cells.

## Abstract (precis)

**Summary:** The authors show a newly identified role of VacA in *Helicobacter pylori* infection through induction of tyrosine phosphorylation of CagA acting through the VacA receptor RPTPα.

## INTRODUCTION

*Helicobacter pylori* is a major causative agent for the development of gastroduodenal diseases, including chronic gastritis, peptic ulcer and gastric cancers (Blaser and Atherton, 2004; Peek and Blaser, 2002). It has been proposed that about 50% of the world's population is infected with *H. pylori*, but only a small number of the infected individuals develop severe clinical manifestations such as gastric adenocarcinoma (Wroblewski et al., 2010). Although a number of virulence factors have been found in *H. pylori*, vacuolating cytotoxin (VacA) and cytotoxin-associated gene A (CagA) are considered to be the major factors in *H. pylori*-induced diseases (Blaser and Atherton, 2004; Peek and Blaser, 2002).

VacA is a potent cytotoxin secreted by most clinical isolates of *H. pylori*, and shows pleiotropic actions in cultured gastric epithelial cells, including generation of vacuoles in the cytoplasm, mitochondrial damage leading to apoptosis, and modulation of signal transduction pathways associated with immune responses (Boncristiano et al., 2003; Hisatsune et al., 2007; Isomoto et al., 2010; Nakayama et al., 2004, 2009; Yamasaki et al., 2006). To facilitate their biological actions in host cells, VacA binds to specific surface receptors. We have identified three different cell surface proteins as VacA receptors: receptor protein tyrosine phosphatase α and β (RPTPα and RPTPβ) and low-density lipoprotein receptor-related protein-1 (LRP1) (Yahiro et al., 1999, 2003, 2012). In addition, other molecules, including sphingomyelin, have been reported to serve as VacA receptors (Gupta et al., 2010). Of these VacA receptors, during *H. pylori* infection, RPTPβ is associated with the development of gastric ulcers in experimental animal models and LRP1 is involved in VacA-dependent autophagy, followed by CagA degradation in infected host cells (Fujikawa et al., 2003; Tsugawa et al., 2012; Yahiro et al., 2012). These data suggest that both receptors are involved in intoxication by VacA. Therefore, we speculate that both receptors, RPTPβ and LRP1, are associated with the development of gastric disorders in *H. pylori* infection. However, the role of RPTPα in intoxication with VacA is unclear.

Previous studies have shown that RPTPα contributes to activation of cellular Src kinase (Src) and other Src family kinase (Su et al., 1999). It has been shown that Src activity is elevated in RPTPα-overexpressing cultured cells, whereas the opposite was observed in RPTPα-deficient cells (den Hertog et al., 1993; Harder et al., 1998; Su et al., 1999; Zeng et al., 2003; Zheng et al., 1992). Furthermore, it has been reported that Src kinase activity is reduced in RPTPα-knockout mice (Harder et al., 1998; Ponniah et al., 1999). Therefore, RPTPα is an important physiological regulator of Src. RPTPα can dephosphorylate both phosphorylated tyrosine residues, pTyr530 and pTyr418 (human Src numbering throughout; the inhibitory phosphorylation site and active site of Src, respectively), thereby causing Src activation following autophosphorylation of Tyr418 (Boggon and Eck, 2004; Vacaru and den Hertog, 2010; Zheng et al., 2000). In addition, based on immunohistochemistry using human gastric cancer tissues, it has been suggested that RPTPα is associated with the progression of gastric cancer (Wu et al., 2006).

In the present study, we show the role of RPTPα in VacA intoxication and also demonstrate that VacA is associated with CagA phosphorylation in AZ-521 cells during *H. pylori* infection. We propose the possibility that VacA induces CagA phosphorylation through RPTPα in AZ-521 cells.

## RESULTS

### VacA induces Src phosphorylation *in vitro*

**Fig. 1.**
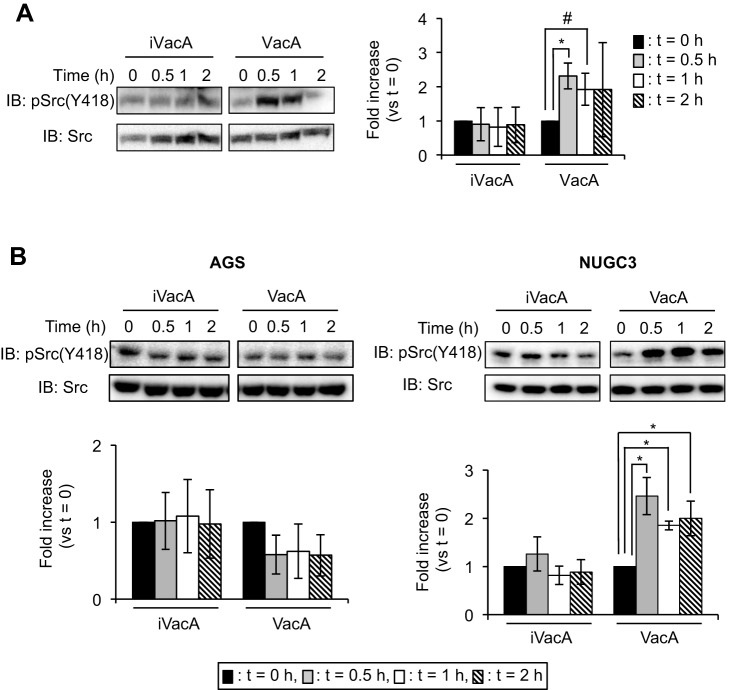
**Detection of phosphorylated Src at Tyr418 induced by VacA.** (A) VacA (120 nM) or heat-inactivated VacA (iVacA) was added to AZ-521 cells followed by incubation at 37°C, 5% CO_2_ for the indicated times. Cells were washed with cold PBS and lysed with SDS sample buffer. Proteins were transferred to membranes and detected using anti-phospho-Src(Tyr418) or anti-Src antibodies. Quantification of signals was normalized to total Src protein. (B) VacA or iVacA was added to AGS and NUGC3 cells and the cultures were incubated at 37°C, 5% CO_2_ for the indicated times. Data were analyzed by two-tailed Student's *t*-test. Results are representative of five independent experiments and data are means±s.d. values from triplicate experiments with an *n*=5 per experiment. *P<0.01; ^#^*P*<0.05 (vs *t*=0). IB, immunoblotting.

Previous studies have shown that VacA modulates signal transduction pathways in AZ-521 cells (Hisatsune et al., 2007; Nakayama et al., 2009). In this study, we found that VacA enhanced phosphorylation at Tyr418 in Src, but heat-inactivated VacA (iVacA) did not have similar effects (Fig. 1A). We also examined the effect of VacA on Src phosphorylation at Tyr418 using other gastric epithelial cells: AGS cells (a human gastric adenocarcinoma cell line) and NUGC3 cells (a gastric cancer cell line). Although VacA stimulated Src phosphorylation at Tyr418 in NUGC3 cells as well as in AZ-521 cells, we did not find any effects of VacA in AGS cells (Fig. 1B), suggesting that Src phosphorylation induced by VacA is dependent on cell type.

### VacA-induced Src phosphorylation is mediated by RPTPα

**Fig. 2.**
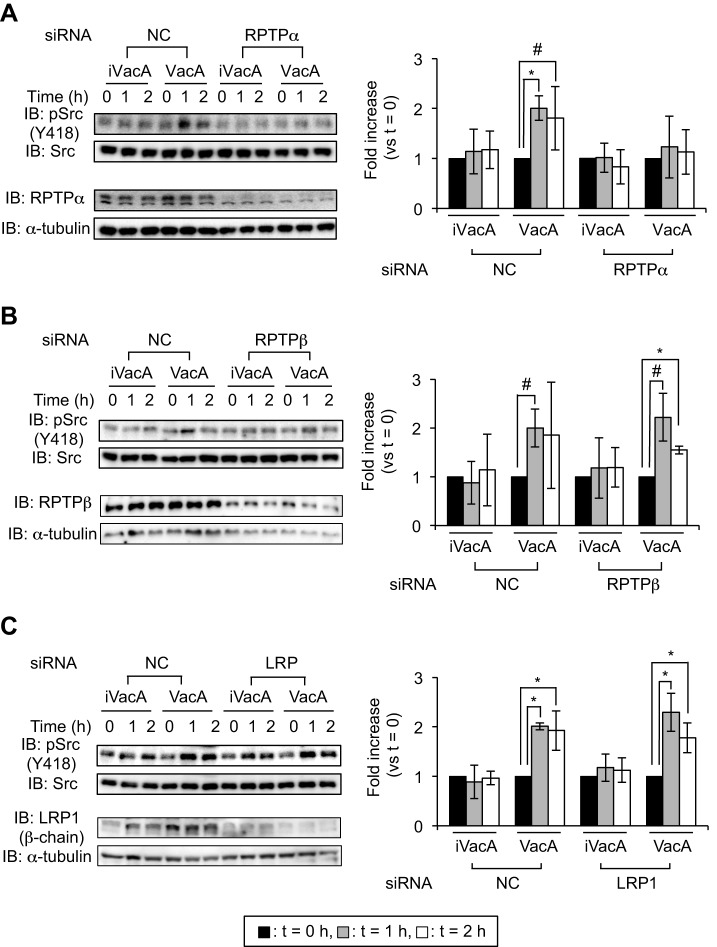
**Detection of phospho-Tyr418 Src in siRNA-transfected AZ-521 cells.** VacA (120 nM) or heat-inactivated VacA (iVacA) was added to siRNA-transfected AZ-521 cells and cells were incubated at 37°C, 5% CO_2_ for the indicated times. Phospho-Tyr418 Src in the presence of VacA in *RPTPα* (A)-, *RPTPβ* (B)- or *LRP1* (C)-siRNA-transfected AZ-521 cells was examined using specific antibodies. Effects of siRNAs were validated by immunoblotting (IB) using specific antibodies. α-tubulin served as a loading control. Signal intensity was normalized to total Src. Data were analyzed by two-tailed Student's *t*-test. Results are representative of five independent experiments and data are means±s.d. values from triplicate experiments with an *n*=5 per experiment. *P<0.01; ^#^*P*<0.05 (vs *t*=0). NC, negative control siRNA.

**Fig. 3.**
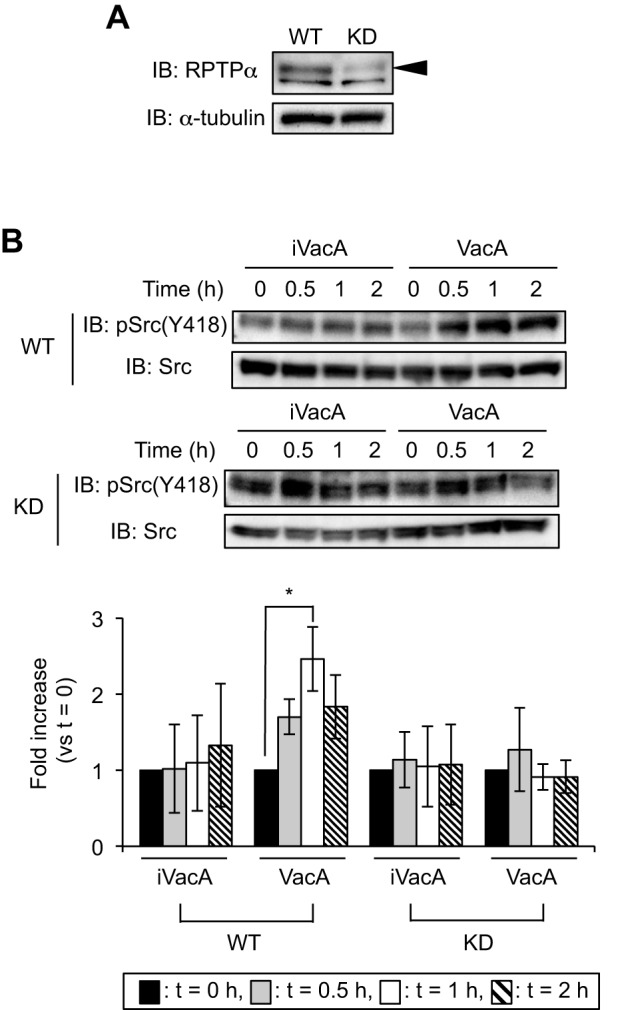
**VacA does not induce phosphorylation of Src in *RPTPα* constitutive-knockdown AZ-521 cells.** (A) Verification of knockdown cells by immunoblotting (IB). Signals were generated using anti-RPTPα antibody. α-tubulin served as a loading control. Arrowhead represents RPTPα. WT, wild-type AZ-521 cells; KD, *RPTPα* constitutive-knockdown AZ-521 cells. (B) Detection of phospho-Tyr418 Src in knockdown cells. Cells were incubated with VacA or iVacA at 37°C, 5% CO_2_ for the indicated times. Signals were generated using anti-phospho-Src(Tyr418) and anti-Src antibodies, and phospho-Src signal intensity was normalized to total Src. Data were analyzed by two-tailed Student's *t*-test. Results are representative of five independent experiments and data are means±s.d. values from triplicate experiments with an *n*=5 per experiment. *P<0.01 (vs *t*=0). IB, immunoblotting.

VacA intoxicates host cells through specific surface receptors. We previously identified three molecules (RPTPα, RPTPβ and LRP1) as VacA receptors (Yahiro et al., 1999, 2003, 2012). To evaluate which of these VacA receptors contributes to Src phosphorylation, we used small-interfering RNA (siRNA)-mediated knockdowns, which targeted each of the VacA receptors, in AZ-521 cells. Although VacA induced phosphorylation at Tyr418 of Src in the presence of control siRNA, VacA did not induce phosphorylation at Tyr418 of Src in siRNA-mediated *RPTPα* knockdown AZ-521 cells (Fig. 2A). On the other hand, in AZ-521 cells, VacA enhanced phosphorylation at Tyr418 in Src after *RPTPβ* or *LRP1* silencing (Fig. 2B,C). To verify these results, we also examined Src phosphorylation induced by VacA using the *RPTPα* constitutive-knockdown AZ-521 cells constructed by a shRNA lentiviral expression system (Yahiro et al., 2012). We found that VacA did not enhance phosphorylation at Tyr418 in Src in *RPTPα* constitutive-knockdown AZ-521 cells (Fig. 3), consistent with the results using the siRNA-mediated *RPTPα* knockdown AZ-521 cells (Fig. 2A). Taken together, we speculate that RPTPα, but not RPTPβ or LRP1, is involved in VacA-dependent phosphorylation at Tyr418 in Src.

### VacA production by *H. pylori* induces CagA phosphorylation and co-immunoprecipitation with SHP2 phosphatase

**Fig. 4.**
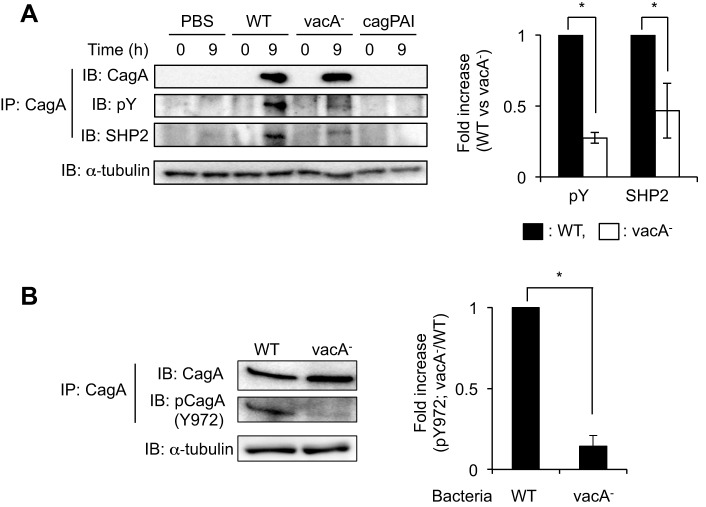
**Detection of pCagA during *H. pylori* infection.** (A,B) Bacteria were allowed to infect AZ-521 cells at 37°C, 5% CO_2_ for 9 h and translocated CagA proteins were recovered by immunoprecipitation (IP) using anti-CagA antibody. To quantify pCagA (pY) or SHP2 phosphatase (A) and pCagA at Tyr972 (Y972) (B), signals were normalized to precipitated CagA protein. α-tubulin served as a loading control. Data were analyzed by two-tailed Student's *t*-test. Results are representative of five independent experiments and data are means±s.d. values from triplicate experiments with an *n*=5 per experiment. WT, *H. pylori* strain ATCC43504; vacA^−^, *vacA* gene-disrupted mutant of ATCC43504; cagPAI, cagPAI-deleted mutant of ATCC43504. *P<0.01 (WT versus *vacA* mutant strain). IB, immunoblotting.

Previous studies have shown that CagA is delivered into gastric epithelial cells directly via a type-IV secretion system and then translocated CagA is tyrosine-phosphorylated by Src family kinases, including Src (Odenbreit et al., 2000; Selbach et al., 2002; Stein et al., 2002). In this study, we have shown that VacA induces phosphorylation at Tyr418 in Src in AZ-521 cells (Figs 1 and 2). We therefore hypothesized that Src phosphorylation induced by VacA is important in CagA phosphorylation during *H. pylori* infection. To evaluate this hypothesis, we examined whether VacA production of *H. pylori* stimulates CagA phosphorylation by challenging cells with wild-type and *vacA* mutant strains in AZ-521 cells. Although total CagA protein, precipitated with anti-CagA antibody, was similar in both wild-type and *vacA* mutant strains, the amount of phosphorylated CagA (pCagA) seen following infection with wild-type *H. pylori* was significantly greater than that seen following infection with a *vacA* mutant strain (Fig. 4A). We next verified the effect of VacA on SHP2 phosphatase, because it has been demonstrated that SHP2 phosphatase specifically binds to pCagA in *H. pylori*-infected host cells (Higashi et al., 2002a). When we carried out immunoprecipitation using anti-CagA antibody following *H. pylori* infection of AZ-521 cells, precipitated SHP2 phosphatase following infection with a *vacA* mutant strain was decreased compared with that seen with wild-type *H. pylori* infection (Fig. 4A). In addition, we also analyzed the effect of VacA on CagA phosphorylation in NUGC3 cells. We found that CagA phosphorylation was stimulated in the early phase (2 h) of infection with wild-type *H. pylori* more than was seen following infection with a *vacA* mutant strain. This was not the case in the late phase of infection (8 h) (Fig. S1).

**Fig. 5.**
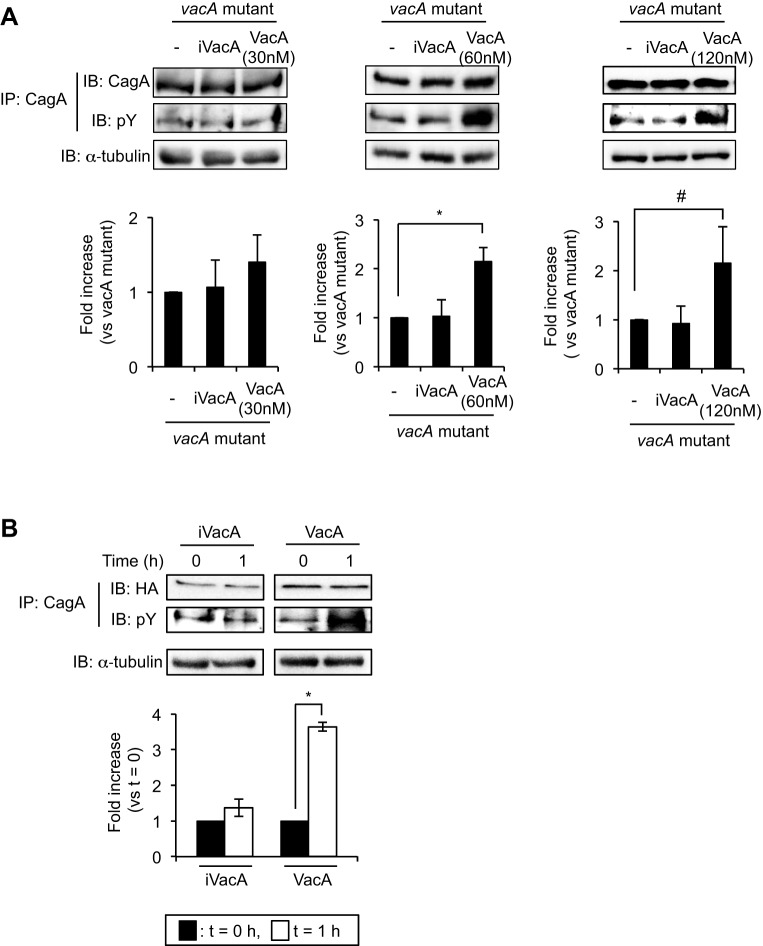
**VacA induces CagA phosphorylation.** (A) Supplementation of purified VacA or heat-inactivated VacA (iVacA) during infection with *vacA* mutant strain. Purified VacA (30, 60 and 120 nM) or iVacA (same concentration with VacA) was added to the *vacA*-mutant-strain-infected AZ-521 cells 7 h post-infection (MOI=100). Cells were further incubated at 37°C, 5% CO_2_ for 2 h. Translocated CagA proteins were precipitated using anti-CagA antibody and signals were detected using anti-phosphotyrosine (pY) and anti-CagA antibodies. α-tubulin served as a loading control. To quantify the amount of pCagA, signals were normalized to precipitated total CagA protein. *P<0.01; ^#^*P*<0.05 (vs *vacA* mutant strain without supplementation). (B) Effect of VacA on CagA phosphorylation in AZ-521 cells expressing HA-tagged CagA (HA). After transfection with a plasmid containing HA-tagged *cagA* gene (Higashi et al., 2002b) in AZ-521 cells, VacA (120 nM) or iVacA was added and cells were incubated at 37°C, 5% CO_2_ for 1 h and then pCagA was precipitated with anti-CagA antibody. Signals were generated using anti-pY and anti-HA-tag antibodies. To quantify pCagA, signals were normalized to HA. Data were analyzed by two-tailed Student's *t*-test. Results are representative of five independent experiments and data are means±s.d. values from triplicate experiments with an *n*=5 per experiment. *P<0.01 (vs *t*=0). IP, immunoprecipitation; IB, immunoblotting.

Several phosphorylation sites in Glu-Pro-Ile-Tyr-Ala (EPIYA) motifs of CagA have been identified. CagA Tyr972 in an EPIYA-C motif is a major Src-phosphorylation site (Backert et al., 2001). A previous study has shown that the EPIYA motif in CagA of wild-type *H. pylori* (strain ATCC43504) is classified as an ABCCC type (Higashi et al., 2002b). To verify the effect of VacA on phosphorylation at Tyr972 in CagA, we used an antibody that recognizes phosphorylated Tyr972 in CagA (Kwok et al., 2007). Infection with wild-type *H. pylori* enhanced phosphorylation at Tyr972 in CagA compared with an infection with the *vacA* mutant strain (Fig. 4B), suggesting that VacA might be specifically associated with phosphorylation at Tyr972 and its corresponding sites within EPIYA-C motifs of CagA in *H. pylori* infection. To demonstrate VacA-induced CagA phosphorylation more clearly, we next tested the effect of different concentrations of VacA supplementation during infection with a *vacA* mutant strain. VacA (60 nM and 120 nM), but not iVacA, supplementation during infection with a *vacA* mutant strain significantly increased the amount of pCagA (Fig. 5A), indicating that VacA (60 and 120 nM) induces CagA phosphorylation. In addition, we also investigated the effect of VacA supplementation using NUGC3 cells during infection with a *vacA* mutant. We found that VacA (120 nM) but not iVacA significantly increased the amount of pCagA in the same manner as seen in AZ-521 cells (Fig. S1). Next, we assessed whether VacA induces CagA phosphorylation by using hemagglutinin (HA)-tagged *cagA* gene-transfected AZ-521 cells using 120 nM VacA to induce the effects of VacA on CagA phosphorylation (Higashi et al., 2002b). In cells expressing HA-tagged CagA, immunoprecipitation using anti-CagA antibody revealed that CagA phosphorylation was enhanced by VacA, but not iVacA (Fig. 5B), supporting our hypothesis that VacA mediates CagA phosphorylation.

### VacA induces CagA phosphorylation in *cagA* gene-transfected cells in an RPTPα-dependent manner

**Fig. 6.**
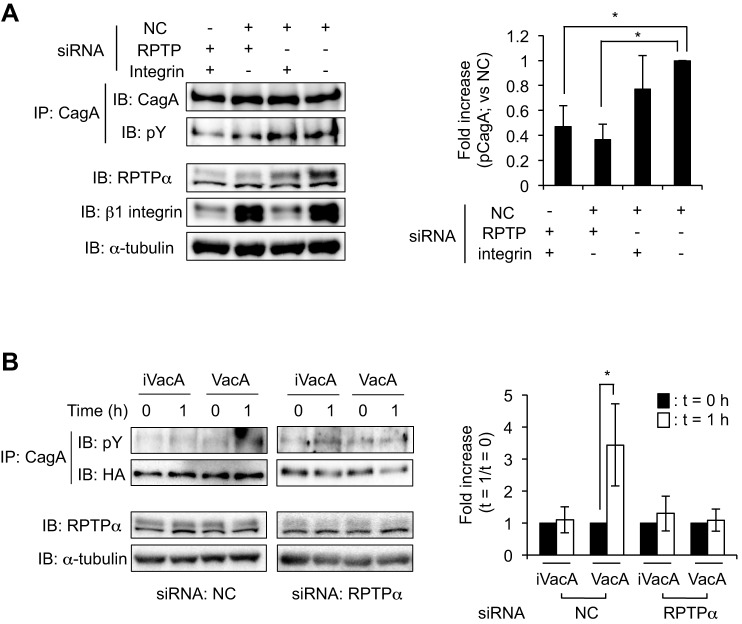
**VacA-induced CagA phosphorylation is mediated through RPTPα.** (A) siRNA-transfected AZ-521 cells were infected with wild-type *H. pylori* strain at 37°C, 5% CO_2_ for 9 h at MOI of 100. Translocated CagA proteins were recovered by immunoprecipitation (IP) using anti-CagA antibody. Signals were generated using anti-phosphotyrosine (pY) and anti-CagA antibodies. To quantify the amount of pCagA, signals were normalized to precipitated CagA protein. The effects of siRNA were validated by western blotting using specific antibodies. α-tubulin served as a loading control. The results are means±s.d. values of five independent experiments. NC, negative control siRNA; RPTP, *RPTPα* siRNA; integrin, *β1-integrin* siRNA. *P<0.01 (vs NC); ^#^*P*<0.05 (vs NC). (B) AZ-521 cells were co-transfected with siRNA and plasmid harboring HA-tagged *cagA* gene and cells were incubated at 37°C, 5% CO_2_ for 24 h. After incubation with 120 nM VacA or heat-inactivated VacA (iVacA), HA-tagged CagA protein was recovered by immunoprecipitation using anti-CagA antibody. Signals were generated using anti-pY and anti-HA-tag antibodies. To quantify the amount of pCagA, signals were normalized to precipitated HA-tagged CagA proteins. RPTPα and α-tubulin served to validate the siRNA and as a loading control, respectively. Data were analyzed by two-tailed Student's *t*-test. Results are representative of five independent experiments and data are means±s.d. values from triplicate experiments with an *n*=5 per experiment. *P<0.01 (vs *t*=0). IB, immunoblotting.

Next, we examined the effect of *RPTPα* silencing on VacA-induced CagA phosphorylation in cells infected with wild-type *H. pylori*. We found that silencing of the *RPTPα* gene with siRNA resulted in a significant reduction of VacA-induced CagA phosphorylation, compared with that in control siRNA-transfected cells. A prior study showed that β1 integrin is associated with CagA translocation in *H. pylori*-infected cells through its interaction with CagL, a component of the type IV secretion apparatus of *H. pylori*, resulting in induction of phosphorylation in both of FAK and Src (Kwok et al., 2007). In addition, they speculated that interaction between CagL and the β1-integrin–FAK–Src signaling pathway was associated with CagA phosphorylation (Kwok et al., 2007). When we examined the effect of *β1-integrin* gene silencing on VacA-induced CagA phosphorylation in cells infected with wild-type *H. pylori*, we found that silencing of the *β1-integrin* gene mediated by siRNA did not result in differences of VacA-induced CagA phosphorylation compared to control siRNA-transfected cells (Fig. 6A), suggesting that RPTPα is responsible for CagA phosphorylation induced by VacA in AZ-521 cells. In addition, we assessed whether RPTPα is crucial for CagA phosphorylation by VacA using AZ-521 cells co-transfected with *RPTPα* siRNA and plasmid harboring an HA-tagged *cagA* gene. We found that VacA did not induce CagA phosphorylation in AZ-521 cells co-transfected with *RPTPα* siRNA and plasmid harboring an HA-tagged *cagA* gene (Fig. 6B). Taken together, these results indicate that RPTPα contributes to the VacA-induced CagA phosphorylation in AZ-521 cells during *H. pylori* infection.

## DISCUSSION

Several VacA receptors have been identified and their roles in the pathogenicity of *H. pylori* have been characterized (Fujikawa et al., 2003; Isomoto et al., 2010; Tsugawa et al., 2012; Yahiro et al., 2012). In this study, we provided new insights into the function of RPTPα in VacA-induced Src phosphorylation in AZ-521 cells (Fig. 1A). When we examined the effects of three VacA receptors, i.e. RPTPα, RPTPβ and LRP1, VacA did not induce Src phosphorylation in siRNA-mediated *RPTPα* knockdown AZ-521 cells, indicating that VacA induces the phosphorylation of Tyr418 in Src through RPTPα, rather than through RPTPβ and LRP1. We therefore propose that binding of VacA to the extracellular domain of RPTPα triggers events leading to the phosphorylation of Tyr418 in Src; this phosphorylation induces subsequent actions in host cells, including CagA phosphorylation. Previous studies have indicated that RPTPα, through its Tyr789 residue, interacts with Src, leading to dephosphorylation of Tyr527 in the C-terminal of Src, autophosphorylation of Tyr418 and activation of Src (Bagrodia et al., 1991; den Hertog et al., 1993; Su et al., 1999; Zheng et al., 1992, 2000). The importance of Tyr789 in RPTPα is unclear because RPTPα lacking Tyr789 is still able to dephosphorylate Src Tyr527 (Vacaru and den Hertog, 2010; Yang et al., 2002). Binding of Src to Ser204 of RPTPα is required for phosphorylation of Src Tyr418 (Vacaru and den Hertog, 2010). Thus, multiple steps involving RPTPα are required for its interaction with Src. RPTPs, including RPTPα, are believed to express their specific functions by binding of the extracellular ligand, followed by the modulation of downstream molecules, including Src (Stoker, 2005). Although extracellular binding molecules to RPTPα have been reported, including VacA (Zeng et al., 1999; Yahiro et al., 2003), interaction mechanisms between those molecules and RPTPα are not yet available. Further studies are needed to determine how VacA affects the interaction between RPTPα and Src.

Previous studies have shown that binding of type-IV secretion systems with β1 integrin or phosphatidylserine plays a key role in the translocation of CagA into AGS cells in *H. pylori* infection. These translocation mechanisms are involved in crucial pathological actions of CagA in infected host cells (Kwok et al., 2007; Murata-Kamiya et al., 2010). Although understanding the molecular mechanisms of CagA phosphorylation in *H. pylori* infection is crucial in order to explain its pathogenicity, these mechanisms are still debated. In the present study, we have shown that CagA phosphorylation in AZ-521 cells was significantly reduced following infection with a *vacA* mutant, compared to infection with wild-type *H. pylori* (Fig. 4A). Furthermore, the amount of immunoprecipitated SHP2 phosphatase that binds to pCagA was reduced following infection with a *vacA* mutant strain in AZ-521 cells, compared to infection with wild-type *H. pylori* (Fig. 4A). Previous studies have shown that SHP2 phosphatase can specifically bind to CagA in a tyrosine-dependent manner at the EPIYA-C and -D motifs (Higashi et al., 2002a; Nagase et al., 2015; Naito et al., 2006). Although several tyrosine phosphorylation sites on CagA EPIYA motifs have been identified, Mueller et al. have proposed that only Src induces phosphorylation at CagA Tyr972, located in the EPIYA-C motif (Mueller et al., 2012). Following this observation, we examined the stimulatory effect of VacA on CagA phosphorylation at Tyr972 in AZ-521 cells infected with wild-type and *vacA* mutant strains. Following infection with wild-type and *vacA* mutant strains, translocated CagA proteins in AZ-521 cells were similar in amount, but phosphorylated Tyr972 in CagA following infection with a *vacA* mutant strain was significantly reduced compared to infection with wild-type *H. pylori* (Fig. 4B). Thus, VacA promotes phosphorylation at Tyr972 in CagA. Therefore, we speculate that reduction of precipitated SHP2 phosphatase following infection with a *vacA* mutant strain is due to the reduction of CagA phosphorylation at Tyr972 in an EPIYA-C motif in AZ-521 cells. As shown in Fig. 5A, supplementation of VacA significantly induced CagA phosphorylation in the *vacA* gene-disrupted mutant, compared with control or with supplementation of iVacA. However, detailed information is currently lacking about the physiological concentration of VacA during *H. pylori* infection or relative concentration at the *H. pylori*-infected host-cell surface. Previous studies have indicated that the concentration of VacA (120 nM) used in this study can induce cell vacuolation and cell death (Yahiro et al., 1997, 2012; Yamasaki et al., 2006). These studies on VacA-dependent cell death are performed in the presence of purified VacA without CagA. In addition, Jain et al. have shown VacA-induced mitochondrial fragmentation, leading to mitochondrial dysfunction and resulting in cell death of AZ-521 cells at 8 h post-infection with *H. pylori* strain (Jain et al., 2011). Thus, these observations indicate that VacA can induce mitochondrial dysfunction, leading to cell death. In this study, we showed that VacA-induced CagA phosphorylation was detected at 2 h post-infection with *H. pylori* strains in NUGC3 cells (Fig. S1). When we also evaluated VacA-induced CagA phosphorylation in AZ-521 cells in more detail, we found that the amount of pCagA at 2 h post-infection with wild-type *H. pylori* was significantly higher than following infection with a *vacA* mutant (data not shown). Therefore, we speculate that VacA-induced CagA phosphorylation in AZ-521 and NUGC3 cells occurs as an early phase of infection prior to VacA-dependent cell death. The VacA concentrations needed to accelerate CagA phosphorylation and detailed molecular mechanisms on VacA functions in host cells during *H. pylori* infection need to be examined.

Many studies on the pathological mechanisms of *H. pylori* have been conducted using several gastric epithelial cell lines, including AGS cells. It has been shown that AZ-521 cells exhibit different biological properties compared with other cell lines that are used in studies of *H. pylori*. However, we tried to use AZ-521 cells because this cell line could be useful for the identification of currently unknown VacA-induced functions owing to its sensitivity to VacA (Radin et al., 2011, 2014). Whether VacA-induced CagA phosphorylation is a cell-specific event remains to be determined. In this regard, in AGS cells, Asahi et al. have shown that VacA is positively involved in CagA phosphorylation in the early phase of *H. pylori* infection (4 h), but Argent et al. have demonstrated the opposite finding, that VacA does not affect CagA phosphorylation (Argent et al., 2008; Asahi et al., 2003). When we also examined the effect of VacA on CagA phosphorylation in NUGC3 cells, in the early phase of infection (2 h) the amount of pCagA following infection with wild-type *H. pylori* increased by more than that seen following infection with a *vacA* mutant. However, in the late phase of infection (8 h) there was no difference between the two treatment groups (Fig. S1), indicating that different levels of VacA-induced CagA phosphorylation are observed in different cell lines. In addition, previous studies have shown that VacA has suppressive effects on the actions of CagA in host cells (Ricci et al., 1996; Tegtmeyer et al., 2009). In one instance, VacA suppressed CagA-dependent cell elongation in AGS and MKN28 cells by inhibition of Erk1/2 activation (Tegtmeyer et al., 2009). However, the action of VacA stimulated the activation of Erk1/2 in other types of cell lines (AZ-521 and TMK1) (Mitsuno et al., 2001; Yahiro et al., 2015). Although the differences in the effects of VacA on CagA responses in each of the cell lines remain unclear, these results indicate that the action of VacA in host cells occurs in a cell-type-specific manner.

**Fig. 7.**
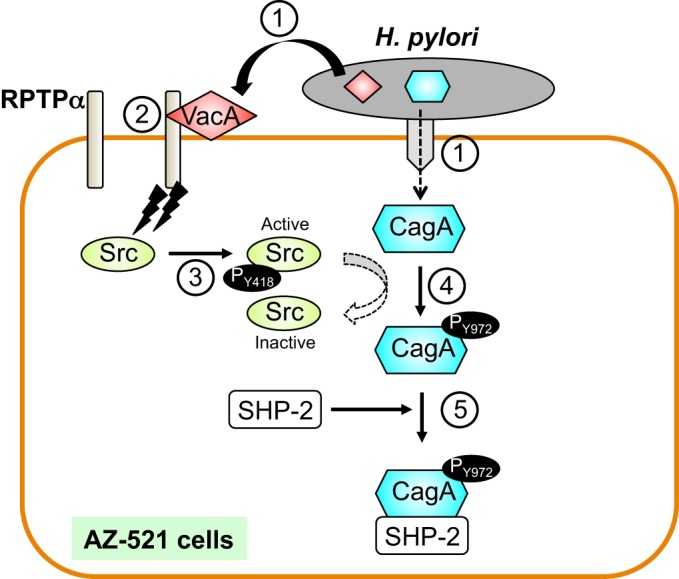
**Putative model showing VacA-induced CagA phosphorylation during *H. pylori* infection in AZ-521 cells.** *H. pylori* injects CagA into gastric epithelial cells by a type-IV secretion system and secretes VacA into the extracellular space (step 1). Secreted VacA binds to the extracellular domain of RPTPα (step 2). VacA induces Src phosphorylation at Tyr418 through RPTPα, resulting in activation of Src (step 3). It has been shown that activated Src promotes tyrosine phosphorylation at Tyr972 of an EPIYA-C motif in translocated CagA and pCagA then inactivates Src (Selbach et al., 2003; Mueller et al., 2012) (step 4). Finally, pCagA interacts with other host molecules, including SHP2 phosphatase (step 5).

In summary, we have shown that VacA promotes CagA phosphorylation through RPTPα in AZ-521 cells (Fig. 7). Our findings demonstrate the possibility that VacA induces CagA phosphorylation in AZ-521 cells during *H. pylori* infection through the VacA-RPTPα-Src signaling pathway. Activation of CagA by a VacA-dependent pathway in AZ-521 cells seems to be important in the pathogenesis seen in *H. pylori* infection.

## MATERIALS AND METHODS

### Mammalian cells and antibodies

Although AZ-521 cells (the Japan Health Sciences Foundation) were recognized as a gastric cancer epithelial cell line, RIKEN BioResource Center recently reported that AZ-521 is a misidentified cell line of HuTu-80, human duodenum carcinoma (http://www.brc.riken.jp/lab/cell/english/urgent_AZ521.pdf) (Yahiro et al., 2015). We also examined our stored AZ-521 cells, which were identified as HuTu-80 cells by short-tandem-repeat analysis [Japanese Collection of Research Bioresources (JCRB), Osaka, Japan]. AZ-521 cells were grown in Eagle’s minimum essential medium (Sigma-Aldrich, St Louis, MO, USA) supplemented with 10% fetal bovine serum (FBS; Nichirei Biosciences, Tokyo, Japan). NUGC3 (JCRB) and AGS (ATCC, Manassas, VA, USA) cells were grown in RPMI-1640 medium (Sigma-Aldrich) supplemented with 10% FBS. Constitutive *RPTPα* knockdown AZ-521 cells were constructed using the pSH1-H1-H1-Puro shRNA lentiviral expression system (SBI Inc., Mountain View, CA, USA) as described previously (Yahiro et al., 2012). Anti-CagA (1:100 and 1:1000; cat# sc-25766) and anti-SH-PTP2 (1:1000; cat# sc-7384) antibodies were obtained from Santa Cruz Biotechnology (Dallas, TX, USA). Anti-phosphotyrosine (1:5000; cat# 9411), anti-Src (1:2000; cat# 2109), anti-β1-integrin (1:2000; cat# 9699), anti-α-tubulin (1:2000; cat# 2512) and anti-HA (1:2000; cat# 2367) antibodies were from Cell Signaling Technology (Danvers, MA, USA). Anti-RPTPβ (1:1000; cat# 610179) and anti-phospho-Src(Tyr418) (1:100 and 1:1000; cat# 44660G) antibodies were obtained from BD Biosciences (San Jose, CA, USA) and Invitrogen (Camarillo, CA, USA), respectively. Anti-RPTPα antibody (1:1000) was prepared as described earlier (De Guzman et al., 2005). Anti-LRP1 antibody (11H4; 1:1000) was kindly provided by Dr D. K. Strickland (University of Maryland). Anti-phosphorylated CagA(Tyr972) antibody (1:2000) was prepared as described previously (Kwok et al., 2007).

### Infection assay

*H. pylori* strain ATCC43504 was used as a parent strain and *vacA* gene-disrupted mutant or whole *cag* pathogenicity island (cagPAI)-deleted mutant (cagPAI) strains were constructed as previously described (Lu et al., 2005). Bacteria were cultured in *Brucella* broth (BD, Franklin Lakes, NJ, USA) supplemented with 10% FBS and 10 µg/ml vancomycin at 37°C for 48 h with shaking under microanaerobic conditions. After preparation, bacteria were added to AZ-521 cells at a multiplicity of infection (MOI) of 100 and were incubated at 37°C, 5% CO_2_ for the indicated times. After incubation, AZ-521 cells were washed twice with cold phosphate-buffered saline (PBS) and the whole cell lysate was prepared as previously described (Asahi et al., 2000).

### Immunoprecipitation

To detect pCagA or pCagA/SHP2 phosphatase complex in the *H. pylori*-infected cells, we performed immunoprecipitation as previously described (Asahi et al., 2000). In brief, proteins were precipitated using anti-CagA antibody (1:100) at 4°C and precipitated proteins were recovered using nProtein A Sepharose 4 Fast Flow (GE Healthcare, Uppsala, Sweden) according to the manufacturer's instructions. The precipitates were washed five times with lysis buffer (50 mM Tris-HCl pH 7.4, 1% Triton X-100, 5 mM EDTA and 1 mM Na_3_VO_4_) and once with 10 mM HEPES buffer (pH 8.0), and then eluted in SDS sample buffer (2% SDS, 10% glycerol, 5% 2-mercaptoethanol, 0.01 mg/ml bromophenol blue, 62.5 mM Tris-HCl pH 6.8).

A plasmid harboring an HA-tagged *cagA* gene (Higashi et al., 2002a,b) was transfected in AZ-521 cells using Lipofectamine 3000 reagent (Life Technologies, Carlsbad, CA, USA) according to the manufacturer's instructions, following which the cells were incubated at 37°C, 5% CO_2_ for 24 h. After transfection, VacA or iVacA was added and cells were incubated at 37°C, 5% CO_2_ for 1 h and then pCagA was precipitated with anti-CagA antibody in the same manner as above.

Eluted proteins were separated on SDS-PAGE and blotted on PDVF membranes (Millipore, Darmstadt, Germany). Signals were detected using the specific antibodies with ECL Prime Western Blotting Detection Reagent (GE Healthcare) and were visualized by LAS-1000 (GE Healthcare). Quantification of each signal was calculated by Multi Gauge V3.0 (GE Healthcare).

### Effects of VacA on the host-cell molecule

VacA toxin was prepared as previously described (Yahiro et al., 1999). In brief, VacA was precipitated from culture supernatant with ammonium sulfate and purified using a column conjugated with anti-VacA antibodies. Purified VacA eluted in the void volume of a gel-filtration column. Therefore, we believe that purified VacA is in the oligomeric form. Prior to addition of VacA to the monolayer, VacA was treated with 1 M HCl to dissociate its oligomeric structure and induce activation (Yahiro et al., 1999). VacA or iVacA was added to monolayers without supplements and the culture was incubated at 37°C, 5% CO_2_ for the indicated times. After incubation, cells were washed twice with cold PBS and lysed with SDS sample buffer. Cell lysates were subjected to SDS-PAGE and proteins were detected using specific antibodies.

### Gene silencing by siRNAs

Knockdown of *RPTPα*, *RPTPβ* or β1-integrin in AZ-521 cells was mediated by specific siRNA (Goel et al., 2005; Müller et al., 2003; Yahiro et al., 2012). Validated *LRP1* siRNA and negative-control siRNA were obtained, respectively, from Life Technologies and Sigma-Aldrich. The indicated siRNA was introduced into AZ-521 cells using Lipofectamine RNAiMax transfection reagent (Life Technologies, Carlsbad, CA, USA) according to the manufacturer's instructions, following which the cells were incubated at 37°C, 5% CO_2_ for 24 h. Expression of target proteins was validated by western blotting using specific antibodies.
